# Evidence‐based use of scalable biomarkers to increase diagnostic efficiency and decrease the lifetime costs of autism

**DOI:** 10.1002/aur.2498

**Published:** 2021-03-08

**Authors:** Thomas W. Frazier, Daniel L. Coury, Kristin Sohl, Kayla E. Wagner, Richard Uhlig, Steven D. Hicks, Frank A. Middleton

**Affiliations:** ^1^ Department of Psychology John Carroll University University Heights Ohio USA; ^2^ Department of Developmental and Behavioral Pediatrics Nationwide Children's Hospital Columbus Ohio USA; ^3^ Department of Child Health University of Missouri Columbia Missouri USA; ^4^ Quadrant Biosciences, Inc. Syracuse New York USA; ^5^ Department of Pediatrics, Division of Academic General Pediatrics Penn State College of Medicine Hershey Pennsylvania USA; ^6^ Department of Neuroscience & Physiology State University of New York Upstate Medical University Syracuse New York USA; ^7^ Department of Pediatrics State University of New York Upstate Medical University Syracuse New York USA

**Keywords:** autism spectrum disorder, biomarkers, cost analysis, early diagnosis, evidence‐based assessment

## Abstract

Challenges associated with the current screening and diagnostic process for autism spectrum disorder (ASD) in the US cause a significant delay in the initiation of evidence‐based interventions at an early age when treatments are most effective. The present study shows how implementing a second‐order diagnostic measure to high risk cases initially flagged positive from screening tools can further inform clinical judgment and substantially improve early identification. We use two example measures for the purposes of this demonstration; a saliva test and eye‐tracking technology, both scalable and easy‐to‐implement biomarkers recently introduced in ASD research. Results of the current cost‐savings analysis indicate that lifetime societal cost savings in special education, medical and residential care are estimated to be nearly $580,000 per ASD child, with annual cost savings in education exceeding $13.3 billion, and annual cost savings in medical and residential care exceeding $23.8 billion (of these, nearly $11.2 billion are attributable to Medicaid). These savings total more than $37 billion/year in societal savings in the US. Initiating appropriate interventions faster and reducing the number of unnecessary diagnostic evaluations can decrease the lifetime costs of ASD to society. We demonstrate the value of implementing a scalable highly accurate diagnostic in terms of cost savings to the US.

**Lay Summary:**

This paper demonstrates how biomarkers with high accuracy for detecting autism spectrum disorder (ASD) could be used to increase the efficiency of early diagnosis. Results also show that, if more children with ASD are identified early and referred for early intervention services, the system would realize substantial costs savings across the lifespan.

## INTRODUCTION

Autism spectrum disorder (ASD) is an etiologically and phenotypically heterogeneous disorder with two symptom domains, social communication/interaction and restricted/repetitive behavior. It is frequently accompanied by co‐occurring medical and mental health conditions and results in variable but lifelong functional impairments and challenges, particularly in social behavior. Over the last two decades, the prevalence of ASD has increased dramatically: in 2000, the prevalence in the US was estimated to be 1 in 150 children, and as recently as 2019, the prevalence was estimated to be 1 in 54 children (Centers for Disease Control and Prevention, 2020).

Caring for an individual with ASD affects all aspects of a family unit, requires considerable use of community resources and results in substantial costs to the family and society (Hyman et al., 2020). The cost of caring for Americans with ASD was estimated to be $268 billion in 2015 (Leigh & Du, 2015), of which $191 billion was attributable to adults with ASD. By 2025, total annual costs are expected to be $461 billion (Leigh & Du, 2015). Medical care represents a significant component of these costs and is 4–6 times higher for individuals with ASD than those without ASD (Shimabukuro et al., 2008).

Early accurate diagnosis followed by appropriate early and intensive intervention has the potential to significantly decrease lifetime cost while improving functioning and well‐being. Randomized controlled trials indicate that a significant proportion of children with ASD who have access to early intensive behaviorally‐based interventions, including naturalistic developmental and behavioral interventions, starting in the 2nd or 3rd year of life (hereafter EIBI for simplicity) show substantial cognitive and functional gains and symptom improvement relative to eclectic or treatment as usual conditions (Dawson et al., 2010; Eldevik et al., 2009; Granpeesheh et al., 2009; Green et al., 2017; Hardan et al., 2015; Howard et al., 2005; Howlin et al., 2009; Kasari et al., 2008; Lovaas, 1987; Mohammadzaheri et al., 2014; National Research Council Division of Behavioral and Social Sciences Education, 2001; Peters‐Scheffer et al., 2011). Associated with these cognitive and functional gains are significant reductions in ongoing costs, including special education and medical care (Dawson & Bernier, 2013).

The current average age of ASD diagnosis in the US is 4 years old (Centers for Disease Control and Prevention, 2019), significantly reducing the ability to positively influence early developmental trajectories. To address the growing need to identify children with ASD early, the American Academy of Pediatrics (AAP) developed and published a Surveillance and Screening Algorithm for ASD (Johnson & Myers, 2007) and in 2020 released an updated clinical report with consistent recommendations for physicians in primary care to screen all children for ASD (Hyman et al., 2020). Despite the clear guidance to universally screen for ASD in primary care settings, fewer than 60% of pediatricians administer an ASD‐specific screening tool at the 18‐ and 24‐month preventative care visit (Siu et al., 2016). Limited screening adherence causes physicians to refer for diagnostic evaluations when symptoms fully manifest at older ages, contributing to a delay in final diagnosis and access to services (Siu et al., 2016). Yet, fully implementing recommendations would only further inundate specialty care clinics and limit access to comprehensive diagnostic evaluation.

After a child is identified as “at risk” using a screening tool, there are significant wait times in clinics due to the abundance of time needed to properly conduct the evaluation per patient (e.g., sometimes multiple visits 2–3 h each). CDC Pathways Survey data from 1420 families of children with ASD reported an average wait time of 3 years between parents' first concerns and receiving a diagnosis of ASD (Oswald et al., 2017).

Given these limitations, scalable, easy‐to‐implement diagnostic measures, used in an evidence‐based medicine framework (Guyatt et al., 2002; Youngstrom et al., 2017) and applied to individuals identified at “elevated risk” by current screening measures, could substantially improve the efficiency of ASD identification. A scalable test would be implemented widely into the healthcare system and would need to be highly feasible, efficiently implemented, and cost‐effective. Differentiation of ASD from non‐ASD cases is also a key consideration for any tool implemented after screening because the majority of false positive screens will be children with other developmental or neuropsychiatric conditions (e.g., anxiety, language/communication disorder, ADHD, oppositional defiant disorder, etc.).

Recent progress investigating a saliva based test (Hicks, Rajan, et al., 2018) and remote eye gaze tracking (Frazier et al., 2018) holds promise in ASD diagnostics. Researchers demonstrated that molecules in saliva are highly accurate in differentiating children with ASD from at‐risk children aged 18 months to 83 months (area under the curve or AUC = 0.88) in a large clinical study (*n* = 451; Hicks, Rajan, et al., 2018). In 2019, this technology was released commercially to use in clinical practice as a diagnostic aid (Geddes, 2020). Similarly, eye tracking measures have shown consistent validity in differentiating ASD and non‐ASD individuals responding to social and nonsocial stimuli (Frazier et al., 2017). Recent studies have supported high levels of validity when measurements are aggregated across stimuli and paradigms (Frazier et al., 2016; Pierce et al., 2011), and a recent remote eye gaze tracking assessment indicated high diagnostic accuracy (area under the curve or AUC = 0.86) when an aggregate index was trained and tested in study of 201 children (91 with ASD and 110 non‐ASD) (Frazier et al., 2018).

**FIGURE 1:**
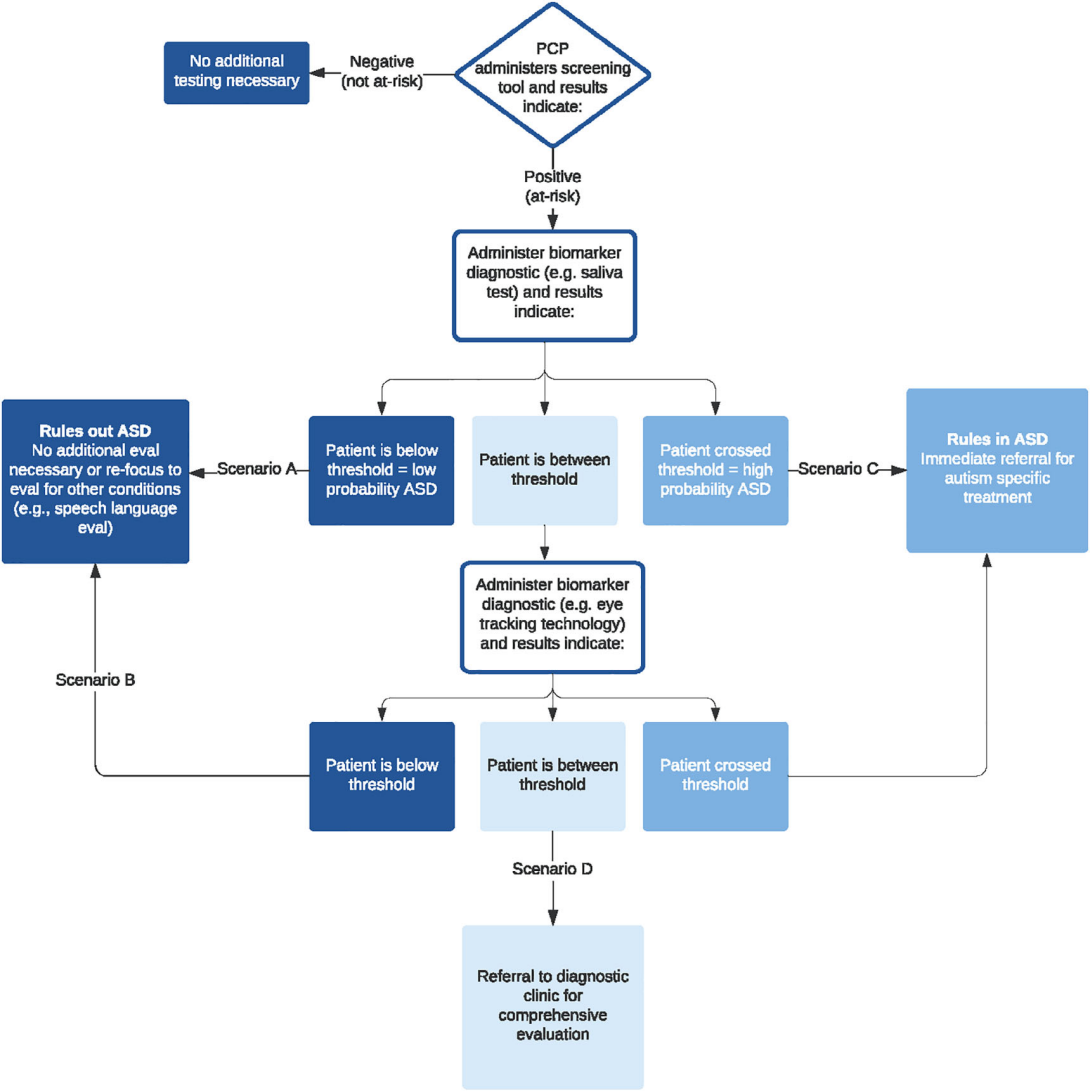
Clinical flow diagram. Results of a biomarker diagnostic determine whether additional evaluation is needed (test/no‐test threshold) and whether treatment might be initiated (treatment threshold). These thresholds are not fixed and are often dependent on additional considerations such as how important it is to identify the condition. Ultimately, exact thresholds are based on the clinical setting and determined by the clinician in consultation with the patient. If the first biomarker results is between thresholds (above the test/no‐test threshold but below the treatment threshold), the second biomarker (whichever was not administered first) would be administered. In this case, Table 5 (see above) would be used to generate the final post‐test probability for use of two biomarkers if results correspond to the presented outcomes (low‐low, high‐high, etc.). However, if some other combination of results were observed, the likelihood ratio values from Tables 1 and 2 could be applied in iterative fashion using Bayes theorem to generate a final post‐test probability based on both biomarkers

The addition of a second order diagnostic aid to the screening process but prior to comprehensive evaluation has the potential to substantially increase the efficiency of ASD identification and lead to more rapid referral for early intervention services. Evidence‐based assessment, including the use of multi‐level diagnostic likelihood ratios, provides a method by which scalable, diagnostic aids with good accuracy could be applied to screen positive cases. Specifically, test scores at levels that decrease the likelihood of ASD could be combined with the postscreening probability to rule out false positives flagged from screening tools. Similarly, test scores at levels that increase the likelihood of diagnosis could be combined with the postscreening probability to identify cases at highest priority and need for evaluation, and, with very high score levels, rule‐in patients with a very high probability of ASD that do not need an expensive and time‐consuming ASD evaluation prior to treatment initiation (Figure 1). Furthermore, parents of children with and without ASD have demonstrated strong interest in an objective diagnostic aid for ASD (Wagner et al., 2019), suggesting that adding an objective diagnostic measure into the current diagnostic process can increase parental acceptance of the diagnosis and decrease the perceived necessity of seeking additional second opinions.

The first aim of the present study is to demonstrate how a scalable diagnostic evaluation tool used in an evidence‐based assessment framework (Guyatt et al., 2002; Sackett et al., 2000) to further triage and inform clinical judgment for high risk cases can substantially improve early ASD identification. Specifically, the paper demonstrates how test scores on scalable diagnostics could be used individually or jointly across a range of postscreening settings to determine whether additional evaluation is needed (test/no‐test threshold) or whether treatment might be initiated (treatment threshold) (Frazier & Youngstrom, 2006; Guyatt et al., 2002; Jenkins et al., 2012; Sackett et al., 2000; Youngstrom et al., 2017). Improvements to the diagnostic process may ultimately lead to a decrease in lifetime costs of ASD by allowing appropriate interventions to be initiated faster and reducing the number of unnecessary diagnostic evaluations. Consequently, the second aim of the present study is to show the cost savings to the US by facilitating an early accurate diagnosis of ASD leading to appropriate early intervention.

## METHODS

### Diagnostic measure validation samples

To illustrate the potential utility of ASD diagnostic biomarkers implemented after primary care screening, two distinct measures were utilized—one based on molecular data (Hicks, Rajan, et al., 2018; Table 1) and a second based on data from remote eye gaze tracking (Frazier et al., 2018; Table 2) to demonstrate to readers that the approach is measure‐agnostic as long as the diagnostic tool being considered is highly scalable and has good validity for ASD and non‐ASD case differentiation. These measures were chosen because they have shown promise in initial development and replication studies (Frazier et al., 2016, 2017, 2018; Hicks et al., 2016; Hicks, Rajan, et al., 2018; Hicks, Uhlig, et al., 2018) and are likely to provide incremental validity by evaluating different neurophysiological processes.

**TABLE 1:** Multi‐level likelihood ratios, post‐test probabilities for base rates .10–.50, and sensitivity and specificity values for the molecular diagnostic measure

Saliva diagnostic (AUC = 0.88)				
Multi‐level likelihood ratios (±95% CI)				
	Very low	Low	High	Very high
Score range	<.25	.26> <.50	.51> <.75	>.76
Likelihood ratio	.16 (.06–.43)	.185 (.08–.41)	7.27 (2.4–21.8)	14.5 (2.0–101.2)
PP (BR = .10)	.02	.02	.45	.62
PP (BR = .20)	.04	.04	.65	.78
PP (BR = .30)	.06	.07	.76	.86
PP (BR = .40)	.10	.11	.83	.91
PP (BR = .50)	.14	.16	.88	.94
Sensitivity	.92	.88	.64	.42
Specificity	.50	.65	.91	.97

*Note*: Sensitivity and specificity values were based on the mid‐point of the range for low, indeterminant, and high score ranges. LR− reported for low and very low score ranges. LR+ reported for high and very high score ranges.

Abbreviations: BR, base rate; PP, posterior probability.

**TABLE 2:** Multi‐level likelihood ratios, post‐test probabilities for base rates .10–.50, and sensitivity and specificity values for the eye‐tracking diagnostic measure

Eye tracking diagnostic (AUC = 0.86)					
Multi‐level likelihood ratios (±95% CI)					
	Very low	Low	Indeterminant	High	Very high
Score range (based on *z*‐score)	<.0	.0> <.70	.70> <1.3	1.3> <2.0	>2.0
Likelihood ratio	.09 (.04–.20)	.39 (.17–.86)	1.47 (.8–2.8)	4.16 (1.6–1.7)	18.30 (5.8–56.4)
PP (BR = .10)	.01	.04	.14	.32	.67
PP (BR = .20)	.02	.09	.27	.51	.82
PP (BR = .30)	.04	.14	.39	.64	.89
PP (BR = .40)	.06	.21	.50	.74	.92
PP (BR = .50)	.08	.28	.60	.81	.95
Sensitivity	.96	.92	.77	.57	.48
Specificity	.60	.68	.88	.95	.97

*Note*: Sensitivity and specificity values were based on the mid‐point of the range for low, indeterminant, and high score ranges. LR− reported for low and very low score ranges. LR+ reported for indeterminant, high, and very high score ranges.

Abbreviations: BR, base rate; PP, posterior probability.

In the first sample, a molecular diagnostic panel was derived and replicated using data from 451 patients (238 children with ASD, 84 children with non‐ASD developmental delay, and 134 neurotypical children). The panel had high overall accuracy for detecting ASD (AUC = 0.88; Hicks, Rajan, et al., 2018). For the purposes of this study, molecular diagnostic data for ASD and all non‐ASD cases are used because presumably a mixture of healthy or neurodevelopmental non‐ASD cases will screen positive on the M‐CHAT‐RF given recent positive predictive value estimates. In the second sample, remote eye gaze tracking to an audio‐visual stimulus was administered using seven distinct social stimulus paradigms (~7‐min total administration). An autism risk index was empirically‐developed and validated for differentiating ASD (*n* = 90) and non‐ASD neurodevelopmental disorder cases (*n* = 110), with high overall accuracy (AUC = 0.86; Frazier et al., 2018).

### Applying evidence‐based assessment

Evidence‐based assessment methods focus on utilizing available research to select the choice of measures and to guide the assessment process. Importantly, evidence‐based assessment allows the clinician to more accurately evaluate test results in relation to the base rate of the condition and the predictive validity associated with a test score. In the present context, the base rate provides the pretest probability of an ASD diagnosis. Given that universal screening is recommended for ASD, that an increasing proportion of children are being screened, and that studies of the M‐CHAT‐RF have suggested positive predictive values as low as 14.6%, the present demonstration assumes a range of initial base rates from .10 (implying 1 in 10 M‐CHAT‐RF screen positives have ASD) to .50 (assuming 1 in 2 M‐CHAT‐RF screen positives have ASD). The upper end of this range is provided to simulate specialty care clinics where base rates of ASD diagnosis often hover around 50% because many patients receive both early screening and additional triage as a result of parental or care provider concern.

In an evidence‐based assessment framework, the predictive validity of a test score is often represented using a likelihood ratio. For tests with continuous or quasi‐continuous scaling, multi‐level likelihood ratios are recommended (Guyatt et al., 2002). Multi‐level likelihood ratios quantify the predictive value of test scores across defined score ranges, in contrast to AUC values, which estimate accuracy across the full range of scores. In the case of ASD, multi‐level likelihood ratios permit the articulation of score ranges that substantively decrease the probability of diagnosis (and in the extreme case may rule it out entirely) as well as score ranges that substantively increase the probability of diagnosis (or in the extreme case rule‐in ASD). Multi‐level likelihood ratios are superior to cut scores because they do not assume that all scores below or above a particular cut score have the same predictive validity. Furthermore, multi‐level likelihood ratios can be easily combined with base rates to understand the probability of diagnosis. Generally, likelihood ratios <.50 are considered useful for decreasing the probability of a condition and those <0.10 are typically strong enough to rule out a diagnosis. Conversely, likelihood ratios >2.0 are considered useful for increasing the probability of a diagnosis and those >10.0 may be strong enough to rule‐in the diagnosis. Likelihood ratios of 1.0 do not alter the probability of diagnosis.

By combining the most likely pretest probability with the likelihood ratio of the observed score, it is possible to generate a post‐test or posterior probability. In an evidence‐based framework, post‐test probabilities can be used to grade clinical decision‐making into more nuanced options and are typically interpreted with reference to two basic clinical decisions—the *test/no test threshold* and the *treatment threshold*. The test/no test threshold defines the probability of the disorder at which further evaluation is recommended and provides a rational approach for determining when to collect additional assessment information. The treatment threshold defines the probability of the disorder at which treatment is recommended. These thresholds are not fixed and are often dependent on a multitude of considerations such as how important it is to identify the condition. For example, the test/no test threshold could be quite low for a condition with a highly accurate, inexpensive, easily scaled, and readily administered test, while the treatment threshold could be very high for expensive, risky treatments. Ultimately, the exact threshold is dependent on the clinician and the clinical setting, and is often set in consultation with the patient. For the present demonstration, we suggest that, in most settings, screening generates at least a 10% post‐screening probability of ASD (in children who screen positive) This probability is assumed to be sufficiently high to pass the test/no test threshold and merit application of scalable, easily‐administered diagnostic measures, particularly given that ASD is often associated with significant lifelong disability and many cases show good response to early intensive intervention. Furthermore, we assume that probabilities below 10% (implying that additional testing has not supported the presence of ASD) are sufficient to rule out additional evaluation.

The treatment threshold is more nuanced and will be highly dependent on the family and patient situation. However, for the purposes of this analysis, we assume that probabilities >50% are sufficient to initiate less intensive, scalable, insurance‐billed treatments such as parent‐mediated intervention (Green et al., 2017) while probabilities >80% are sufficient to rule‐in ASD and initiate therapeutic interventions and de‐prioritize additional expensive diagnostic evaluation (although evaluation for treatment tailoring and other recommendations may ultimately be needed). Finally, we assume that probabilities between 10 and 50% suggest a very high need for additional specialty evaluation, as these individuals are not yet recommended for any intervention, while probabilities between 50 and 80% suggest the next level priority for evaluation because they are recommended to receive less intensive intervention, but may actually require more intensive intervention.

Clinicians could adopt different probability levels or even more nuanced actions. For example, a clinician (in conjunction with the caregiver and patient) may decide that probabilities between .05 and .10 are sufficiently high that additional testing should still be considered, particularly if additional low‐cost, easily‐acquired measures were available. Similarly, if EIBI were to become more widely available and supported through existing funding streams, the post‐test probability level at which intensive treatment could be considered might be substantially lower.

### Diagnostic efficiency analyses

Tables 1 and 2 present diagnostic efficiency statistics, including multi‐level likelihood ratios and posterior probabilities across a range of potential base rates of ASD observed postscreening for the molecular and eye‐tracking diagnostics, respectively. An upper base rate of .50 is provided because this pretest probability is common in specialty care clinics where formal screening has occurred and caregivers have decided to follow through with making and keeping appointments. In this scenario, the molecular and eye‐tracking diagnostic are functioning in the role of specialty care triage, which is analogous to second‐level screening. Multi‐level likelihood ratios were derived for each diagnostic measure using the Evidence‐Based Medicine Toolbox diagnostic test calculator (https://ebm-tools.knowledgetranslation.net/calculator/diagnostic/). For these calculations, score ranges for the molecular and eye tracking diagnostic measures were separately identified that corresponded to “very low,” “low,” “high,” and “very high” proportions of ASD relative to non‐ASD cases based on inspection of the score distributions. For the eye tracking diagnostic, an “indeterminant” category was also used to demonstrate how multi‐level likelihood ratios could account for portions of the score distribution with strong overlap between ASD and non‐ASD cases. This approach has been previously used (Frazier et al., 2007), follows evidence‐based medicine recommendations (Frazier & Youngstrom, 2006; Sackett et al., 2000), and typically generates score ranges with likelihood ratios that are useful for substantially altering the probability of a clinical diagnosis and informing the test/no test and treatment thresholds.

Case examples with low and high scores are presented to demonstrate how biomarkers can be used to facilitate ASD identification, both individually or jointly (through iterative application). AUC values and sensitivity and specificity values at corresponding cut scores are also presented to connect familiar cut score approaches to interpretation.

### Cost savings from accurate early diagnosis and intervention

Using an evidence‐based assessment approach with scalable biomarkers should result in a substantial proportion of children having sufficiently high post‐test probabilities to initiate treatment and a large proportion of children having sufficiently low post‐test probabilities to rule‐out additional evaluation. Thus, cost savings analyses conservatively assume, based on the biomarker accuracy estimates AUC = 0.86–0.88 (Frazier et al., 2018; Hicks, Rajan, et al., 2018), that at least 86% of screened ASD cases will be appropriately triaged. We use 86% in our cost‐savings analysis as indication of how often ASD‐affected children who receive a scalable postscreening diagnostic test will receive appropriate intervention services.

To estimate US cost savings associated with (i) facilitating an early accurate diagnosis of ASD and (ii) early identification leading to appropriate early intensive behavioral interventions, an analysis was conducted to forecast estimates of costs associated with (a) special education (including federal, state and local district expenses for special education) and (b) medical and residential care expenses (including federal and state Medicaid expenses).

This cost–benefit analysis acknowledges the following:

1. Predictors of reduced severity of ASD symptoms as a result of EIBI include age at intervention enrollment, cognitive functioning, and initial ASD symptom severity (Landa, 2018).
2. The proportion of children who become functionally indistinguishable from their peers is probably lower than the proportion often reported in the literature (just under 50% [Lovaas, 1987]). Among children with ASD who receive competently delivered EIBI, between 20 and 50% will be functionally indistinguishable from age‐matched peers; between 20 and 40% will achieve meaningful but moderate gains; and 10–40% will continue to require intensive special education and adult services. For this financial model, we use the results of a meta‐analysis and assume that 29% achieve age‐appropriate functional behavior, 34% achieve meaningful but moderate gains, and 37% require intensive special education and adult services (Peters‐Scheffer et al., 2012).
3. Without EIBI the majority of children with ASD will manifest enduring dependency on special education and adult developmental disability services: among children with ASD who have not received EIBI, a meta‐analysis suggests that only 11% will achieve age appropriate functional behavior, 8% will achieve meaningful but moderate gains, and 81% will require intensive special education and adult services (Peters‐Scheffer et al., 2012).

For these reasons, this cost–benefit analysis is framed in terms of marginal gains as well as the attainment of age appropriate functional behavior.

Additional assumptions in this analysis include the following:

1. Children who are diagnosed with ASD have access to EIBI services. Children who are not identified early as having ASD could still receive interventions; however, these interventions would either be nonspecific to the treatment of core symptoms of autism (e.g., occupational therapy, physical therapy), and/or would be related but balanced between the EIBI and no EIBI scenarios (e.g., speech therapy), and/or would likely be much lower intensity.
2. The costs for EIBI services is assumed to be a representative average for both center‐based and home‐based services (average of $45,000/year, given that children with ASD receive 20–40 h/week of EIBI (Reichow et al., 2018), we used 30 h/week and 50 weeks/year as a conservative estimate and assumed $30/hour (the average hourly rate of a board certified behavior analyst)).
3. The average duration of EIBI is assumed to be 3 years (Jacobson et al., 1998).
4. Consistent with prior literature estimates, 31% of children with ASD are assumed to also have an intellectual disability (ID) (Centers for Disease Control and Prevention, 2014).
5. Children with ASD who achieve age‐appropriate functional behavior are assumed to use family support services only during participation in EIBI; those who make moderate gains or realize minimal effects are assumed to use 18 years of services.
6. All savings shown are net of the expense of providing EIBI (which is assumed to be a medical expense).
7. Children with ASD who ultimately become functionally indistinguishable from their peers are assumed to participate in regular education and have normal medical expenses thereafter; those who make moderate gains are assumed to participate in special education (Peters‐Scheffer et al., 2012) and have medical expenses associated with ASD children who have other comorbid conditions(Peacock et al., 2012); and children who make minimal gains are assumed to participate in intensive special education (Peters‐Scheffer et al., 2012) and have medical expenses associated with ASD children who have other comorbid conditions including intellectual disabilities (Peacock et al., 2012).
8. Cost estimates which include the adult years are made only to age 54, consistent with the average age of mortality in ASD (Hirvikoski et al., 2016). This assumption is conservative since there is a high likelihood that future generations will live beyond 54 with improved medical care and awareness of ASD.
9. Cost estimates are based on the article by Buescher et al., 2014 (Buescher et al., 2014): for Medical and Residential Care, see Table 3; for special education and intensive special education for preschool children (ages 2–5), 2012 cost estimates were $31,460, and $62,920, respectively; for special education and intensive special education for school age children (ages 6–21), 2012 cost estimates were $13,980 and $27,961, respectively. Resent costs (year 2020) were derived from historic cost estimates (year 2012), using annual rates of inflation for Medical Services or Elementary and High School Tuition and Fees, as appropriate (see Table 4).
10. Calculated present‐day costs are assumed to increase annually at the prior 10‐year average annual rate of inflation for Medical Services or Elementary and High School Tuition and Fees, as appropriate (see Table 4).
11. Future costs are discounted to present value at a rate equivalent to the 30‐year US Treasury yield (1.56% on March 5, 2020; U.S. Department of the Treasury, 2020).

**TABLE 3:** Annual medical and residential care cost estimates for individuals with autism spectrum disorder

	Marginal costs without intellectual disability			Marginal costs with intellectual disability		
	Starting ages (years)			Starting ages (years)		
Cost category (year 2012)	2	6	18	2	6	18
Residential care (Medicaid)	$952	$4758	$18,080	$1903	$9516	$36,161
Medical services (excluding autism‐specific behavioral therapies)	$6467	$9053	$13,580	$12,933	$18,106	$27,159

**TABLE 4:** Annual rates of inflation

Year	Elementary and high school tuition and fees (%)	Medical services (%)
2000	6.90	4.29
2001	6.49	4.79
2002	6.92	5.07
2003	6.65	4.47
2004	6.73	5.02
2005	6.54	4.78
2006	5.81	4.14
2007	5.52	5.33
2008	5.77	4.24
2009	5.22	3.21
2010	4.01	3.50
2011	3.78	3.06
2012	3.57	3.90
2013	3.66	3.10
2014	3.89	2.38
2015	3.86	2.45
2016	3.51	3.91
2017	3.59	2.43
2018	4.12	2.17
2019	3.43	3.31
Inflation estimate (10‐year average)	3.74	3.02

*Note*: Source: Consumer Price Index, Bureau of Labor Statistics.

### *Using the assumptions outlined above, cost savings estimates were derived using the specific methodology detailed in* Supporting Information

The present value of cost savings derived from this analysis are apportioned between federal, state and local taxpayers using the following methodology:

1. According to a CSEF Report on State Special Education Finance Systems, support for special education programs is provided by approximately 45% from states, 46% from local districts, and 9% through federal IDEA funding (Dragoo, 2018; Parrish et al., 2003)
2. Medicaid and CHIP cover about half (47%) of American children with special health care needs (Musumeci & Chidambaram, 2019)
3. The federal government contributes at least $1 in matching funds for every $1 a state spends on Medicaid. The fixed percentage the federal government pays, known as the “FMAP,” varies by state, with poorer states receiving larger amounts for each dollar they spend than wealthier states. The national average of 76.5% (KFF, 2020) was used in this analysis.

Avalere Health conducted an independent examination of the underlying assumptions associated with this cost savings analysis (included in Supporting Information).

## RESULTS

### Improving ASD identification

For both diagnostic measures, low and very low scores show good sensitivity, while high and very high scores show good specificity. However, sensitivity and specificity values do little to guide clinical judgment as the clinician needs to know what the probability of ASD diagnosis is after utilizing one or both of these measures. This requires the application of likelihood ratios under realistic base rate conditions. As shown in Tables 1 and 2, both the molecular and eye tracking diagnostics have multi‐level LRs that fall in the clinical useful ranges for decreasing (LR <.50) and increasing (LR >2.0) the probability of diagnosis. For example, both measures individually produce very low posterior probabilities when very low scores are observed, even under the postscreening high base rate scenario. Thus, very low scores on either measure are likely sufficient to rule out ASD and either avoid additional testing or re‐focus the priority for evaluation on other issues (e.g., speech language evaluation; see Figure 1 scenario A). While low scores on either measure alone are insufficient to rule out ASD (except in the lowest base rate conditions BR <.20), jointly observing low scores on these measures is sufficient, even under the highest base rate condition, to rule out ASD and avoid additional testing (Table 5). In Figure 1, this scenario is represented by the path where the first biomarker produces a between threshold result but administration of the second biomarker results in a rule out (middle branch followed by left‐sided branch—scenario B).

**TABLE 5:** Post‐test probabilities across base rates .10–.50 for very low, low, high, and very high molecular and eye‐tracking diagnostic score pairings

	Very low—Very low scores	Low—Low scores	High—High scores	Very high—Very high scores
Molecular likelihood ratio	.16	.19	7.27	14.50
Eye‐tracking likelihood ratio	.09	.39	4.16	18.30
PP (BR = .10)	<.01	<.01	.77	.97
PP (BR = .20)	<.01	.02	.88	.99
PP (BR = .30)	<.01	.03	.93	.99
PP (BR = .40)	.01	.05	.95	.99
PP (BR = .50)	.01	.07	.97	>.99

*Note*: Score combinations were chosen to represent extreme and middle combinations (absent indeterminant values for the eye tracking diagnostic measure). For score combinations, likelihood ratios are used in an iterative fashion and assuming no substantive correlation (*r* <.40) between the molecular diagnostic and the eye tracking diagnostic. If the actual correlation between two measures used iteratively is higher, posterior probabilities will be inflated.

Very high scores on either measure generate post‐test probabilities indicating it is more likely than not (PPs >.60 in all base rate conditions) that the patient being evaluated has ASD. Given the potential benefit and low risks of early behavioral intervention approaches, this information is likely sufficient to recommend initiating treatment in many circumstances (scenario C). At minimum, this information could be used to prioritize individuals for specialty care evaluation (scenario D). High scores on one measure are probably not sufficient to assume ASD is present and recommend expensive interventions, but may be sufficient to recommend less expensive approaches such as parent‐mediated intervention or outpatient social skills training. However, high scores on both measures yield posterior probabilities that suggest EIBI should be initiated (PPs ≥.77 in all base rate conditions), if deemed clinically appropriate for the child (Table 5).

Overall, under most realistic base rate conditions, even when assuming only one postscreening diagnostic measure can be used, many evaluated cases are likely to have posterior probabilities that either rule out ASD or that increase the probability of ASD sufficiently that recommending intervention is warranted, substantially reducing the number of cases that require a specialty care evaluation and improving prioritization of the remaining cases.

### Cost savings from second order diagnostic aid

**FIGURE 2:**
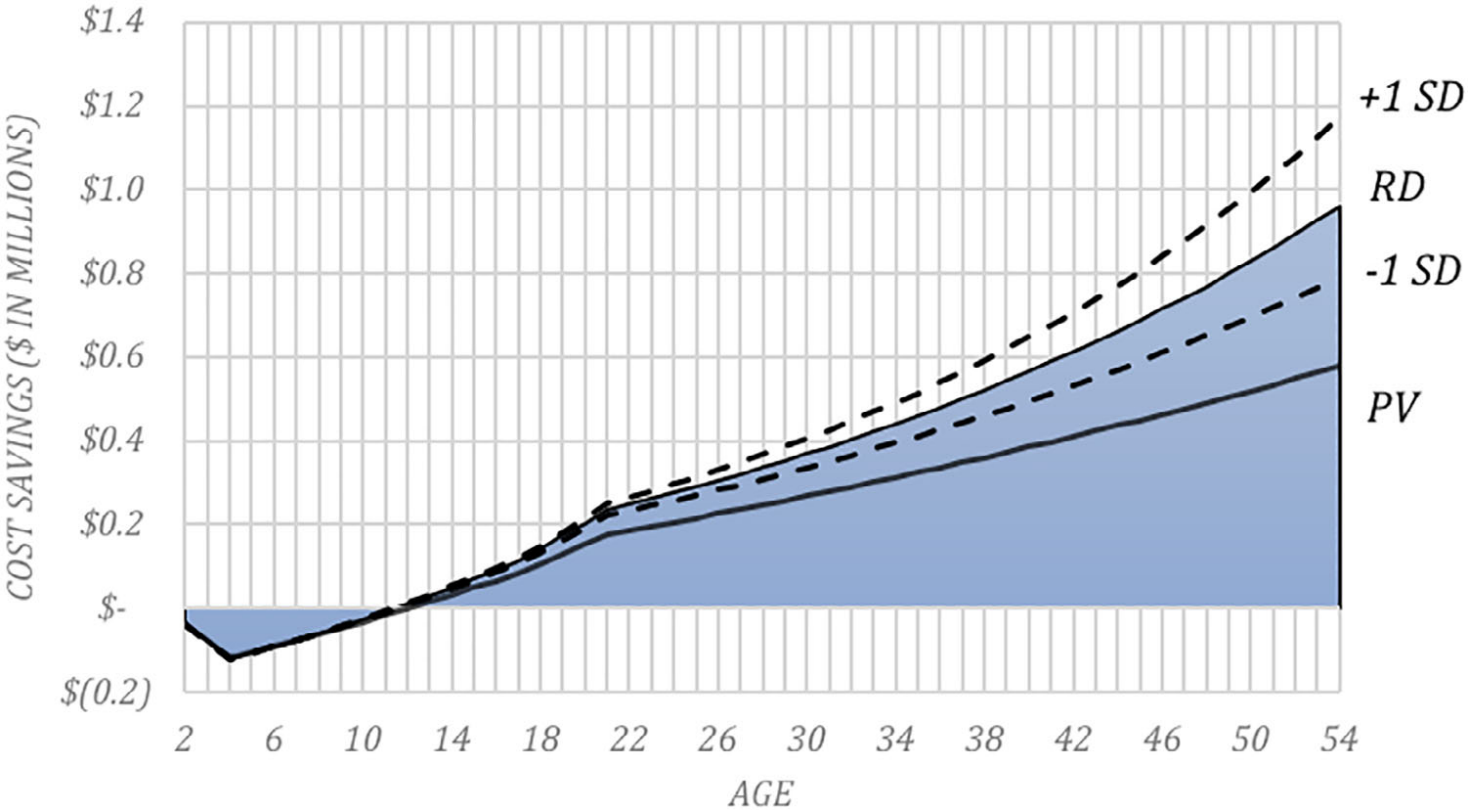
Average cumulative cost savings per ASD child. Average cumulative cost savings per ASD child. The blue shaded area provides an estimate of the cumulative cost savings (or outlays) in real dollars (RD) for each ASD child who experiences early intervention as a consequence of early detection. The dotted lines indicate the same amount adjusted for different inflation estimates across time (±1 standard deviation). The solid line indicates the present value (PV) of real dollar savings (or outlays) experienced through the age indicated. Note that there is a net positive savings in both real dollar and present value terms beginning at age 11 years

In prior research, the cost differential estimated over the lifetime for an ASD child relative to a neurotypical child ranges from $1.4 to $2.4 million per child (Buescher et al., 2014). Due to a range of improvements resulting from EIBI, the societal cost savings in (i) special education and (ii) medical and residential care associated with the recommended changes are estimated to average nearly $580,000 per ASD child (after accounting for a projected accuracy of at least 86% for each of the newly developed eye‐tracking and molecular diagnostic tools); as seen in Figure 2, with approximately 65,000 new children diagnosed as ASD each year in the United States, this cost savings totals over $37 billion/year. Annual cost savings in education exceeds $13.3 billion, with savings of approximately $1.2 billion, $6.0 billion, and $6.1 billion achieved by federal, state and local school districts, respectively. Annual cost savings in medical and residential care exceeds $23.8 billion, with savings of approximately, $8.5 billion and $2.6 billion in Federal Medicaid and State Medicaid spending, respectively. Variations of key model parameters (high and low estimates) were used to estimate the sensitivity of cost savings to different inputs. Results of this sensitivity analysis indicate that even under the most conservative conditions, substantial costs savings are achieved in education (the most conservative model estimates over $5.6 billion saved). Not surprisingly, less conservative estimates yield even greater savings (the least conservative model estimates over $24.6 billion saved). This holds true for costs associated with Medicaid as well (the most conservative model estimates $3.0 billion saved; the least conservative model estimates $19.3 billion saved; see Table S1).

## DISCUSSION

The current ASD screening and diagnostic process is very inefficient, causing large proportions of children with ASD to experience a delay in diagnosis and miss an opportunity to initiate treatment at an early age when it is most effective. There are significant wait times for diagnostic evaluations, contributing to a large gap of time between when children are first identified as “at risk” using screening methods to when interventions are initiated (average age of diagnosis in the US is 4 years old).

The present study demonstrates that using scalable diagnostic measures coupled with an evidence‐based assessment framework could substantially improve ASD identification by: (i) ruling out, de‐prioritizing, or re‐focusing the type of evaluation needed for likely non‐ASD cases with low post‐test probabilities, (ii) identifying cases at the highest need for specialty care evaluation (reducing wait lists for these clinics), (iii) immediately initiating low cost interventions for ASD cases with moderate to high probability, and (iv) immediately initiating EIBI for very high probability cases (Figure 1). While accuracy remains the most important feature to consider when assessing new diagnostic tests, several other strengths suggest that these measures will translate well into a clinical setting, including: rapid administration (<10 min), noninvasive testing methods, results containing quantitative information that is objective and not influenced by rater perceptions, inexpensive equipment, and applicability to a wide range of ages and symptom severities. Parents are often unsatisfied with the measures currently used to evaluate ASD and in many cases, parents do not accept the diagnosis, creating a barrier to access treatment (Crane et al., 2015; Makino et al., 2017).

Secondarily, this study conducted a cost analysis to estimate savings associated with the use of evidence‐based, postscreening diagnostic tools. Results indicated that substantial cost savings are achievable. Specifically, cost/benefit analysis yielded three major findings: (1) the present value of lifetime societal cost savings in special education, medical and residential care associated with implementing one or more scalable postscreening diagnostic measures is estimated to average nearly $580,000 per ASD child; (2) with approximately 65,000 new children diagnosed with ASD each year in the US, annual cost savings in education would exceed $13.3 billion and annual cost savings in medical and residential care would exceed $23.8 billion; and (3) a total of more than $37 billion/year would be saved when combining cost‐savings for education, medical and residential care. In the first several years following implementation, costs savings may significantly exceed these estimates due to existing pent‐up demand for ASD diagnostic services. More importantly, these changes will positively impact the quality of life for ASD children and their families. To date, approximately one‐fourth of children under age 8 with ASD go undiagnosed, most of which belong to a minority population or are children living in census tracts with lower socioeconomic development, having reduced access to appropriate diagnostic services and therefore not receiving the support they need (Durkin et al., 2017; Wiggins et al., 2020); these children and families will likely benefit most from the recommended changes.

Here, we demonstrate the positive clinical and financial influences that implementing an ASD diagnostic measure can have, reinforcing the need to continue development of highly accurate, scalable biomarkers for ASD. Once promising measures are identified, funding mechanisms are needed to ensure that these tools can be clinically‐ implemented and that widespread adoption can be achieved even in low resource settings. As these tools are validated and widely implemented, it will be key to incorporate them into the existing ASD identification processes and practice guidelines. The present results also emphasize the need to make early intervention programs, including less intensive and cost‐effective parent‐mediated interventions, as well as more‐intensive and costly EIBI and developmental and behavioral intervention packages, more widely available. Rapid progress in scalable biomarker identification and validation, including one measure that is already commercially available and several that are likely to become available in the coming years, means that many more children with ASD will receive an early accurate diagnosis. This deluge of early diagnosis will only be useful if individuals can receive appropriately calibrated early interventions.

While Medicaid and private medical insurance may pay for some portion of EIBI (this varies by insurer and by state), access to EIBI remains dependent on ASD symptoms being identified early, and financial access to these services is often delayed pending a clinical ASD diagnosis. Incorporating a scalable diagnostic measure into the ASD diagnostic process to allow more children with ASD to be identified in early childhood when treatment is most efficacious is a goal consistent with that of Early and Periodic Screening, Diagnostic, and Treatment (EPSDT), a child health service of Medicaid for beneficiaries under age 21, that requires coverage for all health care services (e.g., preventative and treatment services) that are found to be medically necessary to “discover and treat childhood health conditions before they become serious or disabling” (California's Healthcare Foundation, 2015; Center for Medicaid & Medicare Services, 2014; Medicaid.gov., 2019). In the fiscal year 2014, over 40 million children were eligible for EPSDT, suggesting a strong need for validated scalable diagnostic measures to aid identification of a significant number of children in the US with ASD.

### Limitations and future directions

Demonstration of an evidence‐based assessment approach was intentionally presented in a measure‐agnostic fashion, so that any well‐validated, scalable diagnostic measures for ASD could be applied using this framework. We chose two measures that were developed and validated in large samples, but also encourage all emerging ASD diagnostics, including the two used as examples, to continue to build predictive validity evidence, particularly across settings and sub‐populations. It is crucial that ASD diagnostic biomarkers be validated in the most stringent fashion by comparison to both healthy controls and to non‐ASD developmental disability or developmental neuropsychiatric controls who often mimic ASD presentations and frequently screen positive during the initial screening process. Furthermore, validation should examine low resource and race/ethnic sub‐populations to ensure that existing validity evidence appropriately generalizes. And, finally, we would not suggest using even well‐validated ASD biomarkers without first implementing the recommended first‐level questionnaire screening tools as these tools provide an inexpensive and rapid method for altering the post‐test probability prior to a more expensive or time‐consuming biomarker data collection. Instead, it is optimal if biomarkers are used only for screen‐positives and within the context of evidence‐based evaluation to leverage existing screeners.

The cost–benefit analysis also makes several assumptions—many consistent with prior cost savings analyses (Ganz, 2007; Leigh & Du, 2015). It is important to note that even if fewer ASD cases are accurately identified than assumed, cost savings remains substantial. Furthermore, modest variations in assumptions were found not to impact the overall message that early accurate identification followed by effective early intervention will generate massive savings.

In conclusion, application of scalable ASD diagnostic biomarkers using an evidence‐based assessment framework is likely to substantially enhance early ASD identification and provide support for more nuanced clinical recommendations. As a result of substantially improved early ASD identification, substantial lifetime costs savings can be realized as a result of a greater proportion of ASD‐affected children receiving appropriate early intervention.

## CONFLICT OF INTEREST

Thomas W. Frazier is the former Chief Scientific Officer of Autism Speaks and developer of the eye tracking test that was used in the EBM analysis. Thomas W. Frazier has received federal funding or research support from, acted as a consultant to, received travel support from, and/or received a speaker's honorarium from Quadrant Biosciences, Impel NeuroPharma, F. Hoffmann‐La Roche AG Pharmaceuticals, the Cole Family Research Fund, Simons Foundation, Ingalls Foundation, Forest Laboratories, Ecoeos, IntegraGen, Kugona LLC, Shire Development, Bristol‐Myers Squibb, Roche Pharma, National Institutes of Health, and the Brain and Behavior Research Foundation and has an investor stake in AutismEYES LLC. Steven D. Hicks and Frank A. Middleton are co‐developers of the saliva RNA based autism test that is used in the EBM analysis, and members of the Clinical and Scientific Advisory Boards of Quadrant Biosciences. Kristin Sohl is director of ECHO Autism and both Kristin Sohl and Daniel L. Coury are a member of the Clinical Advisory Board of Quadrant Biosciences. Daniel L. Coury has received federal funding or research support from National Institutes of Health, GW Biosciences, Neurim, Stemina Biosciences, and Stalicla SA; and acted as a consultant to BioRosa, Cognoa, GW Biosciences, and Stalicla SA. Kayla E. Wagner and Richard Uhlig are employees of Quadrant Biosciences. Quadrant Biosciences holds patent rights and exclusive sales rights for the Clarifi ASD saliva test.

## Supporting information


**Appendix S1**: Avalere Health Validation

**Appendix S2**: Cost Savings Analysis Methodology

**Appendix S3.** Supporting Information.

Supplemental Table 1

## References

[aur2498-bib-0001] Buescher, A. V. , Cidav, Z. , Knapp, M. , & Mandell, D. S. (2014). Costs of autism spectrum disorders in the United Kingdom and the United States. JAMA Pediatrics, 168(8), 721–728. 24911948 10.1001/jamapediatrics.2014.210

[aur2498-bib-0002] California's Healthcare Foundation . (2015). An examination of medicaid's coverage determination policies.

[aur2498-bib-0003] Center for Medicaid & Medicare Services . (2014). Clarification of medicaid coverage of services to children with ASD. Retrieved from https://www.medicaid.gov/federal-policy-guidance/downloads/cib-07-07-14.pdf

[aur2498-bib-0004] Centers for Disease Control and Prevention . (2014). Autism and developmental disabilities monitoring (ADDM) network. Retrieved from https://www.cdc.gov/ncbddd/autism/addm.html

[aur2498-bib-0005] Centers for Disease Control and Prevention . (2019). Spotlight on: Delay between first concern to accessing services. Retrieved from https://www.cdc.gov/ncbddd/autism/addm-community-report/delay-to-accessing-services.html

[aur2498-bib-0006] Centers for Disease Control and Prevention . (2020). Data & statistics on autism spectrum disorder. Retrieved from https://www.cdc.gov/ncbddd/autism/data.html

[aur2498-bib-0007] Crane, L. , Chester, J. W. , Goddard, L. , Henry, L. A. , & Hill, E. (2015). Experiences of autism diagnosis: A survey of over 1000 parents in the United Kingdom. Autism, 20(2), 153–162. 10.1177/1362361315573636 25810370

[aur2498-bib-0008] Dawson, G. , & Bernier, R. (2013). A quarter century of progress on the early detection and treatment of autism spectrum disorder. Development and Psychopathology, 25, 1455–1472. 24342850 10.1017/S0954579413000710

[aur2498-bib-0009] Dawson, G. , Rogers, S. , Munson, J. , Smith, M. , Winter, J. , Greenson, J. , Donaldson, A. , & Varley, J. (2010). Randomized, controlled trial of an intervention for toddlers with autism: The early start Denver model. Pediatrics, 125(1), e17–e23. 10.1542/peds.2009-0958 19948568 PMC4951085

[aur2498-bib-0010] Dragoo, K. E. (2018). The individuals with disabilities education act (IDEA) funding: A primer. CRS Report R44624, Version 4. Updated. Congressional Research Service

[aur2498-bib-0011] Durkin, M. S. , Maenner, M. J. , Baio, J. , Christensen, D. , Daniels, J. , Fitzgerald, R. , Imm, P. , Lee, L. C. , Schieve, L. A. , Van Naarden Braun, K. , Wingate, M. S. , & Van Naarden Braun, K. (2017). Autism spectrum disorder among US children (2002–2010): Socioeconomic, racial, and ethnic disparities. American Journal of Public Health, 107(11), 1818–1826. 10.2105/AJPH.2017.304032 28933930 PMC5637670

[aur2498-bib-0012] Eldevik, S. , Hastings, R. P. , Hughes, J. C. , Jahr, E. , Eikeseth, S. , & Cross, S. (2009). Meta‐analysis of early intensive behavioral intervention for children with autism. Journal of Clinical Child and Adolescent Psychology, 38(3), 439–450. 10.1080/15374410902851739 19437303

[aur2498-bib-0013] Frazier, T. W. , Klingemier, E. W. , Beukemann, M. , Speer, L. , Markowitz, L. , Parikh, S. , Wexberg, S. , Giuliano, K. , Schulte, E. , Delahunty, C. , Ahuja, V. , Eng, C. , Manos, M. J. , Hardan, A. Y. , Youngstrom, E. A. , & Delahunty, C. (2016). Development of an objective autism risk index using remote eye tracking. Journal of the American Academy of Child & Adolescent Psychiatry, 55(4), 301–309. 10.1016/j.jaac.2016.01.011 27015721 PMC4808563

[aur2498-bib-0014] Frazier, T. W. , Klingemier, E. W. , Parikh, S. , Speer, L. , Strauss, M. S. , Eng, C. , Hardan, A. Y. , & Youngstrom, E. A. (2018). Development and validation of objective and quantitative eye tracking‐based measures of autism risk and symptom levels. Journal of the American Academy of Child & Adolescent Psychiatry, 57(11), 858–866. 10.1016/j.jaac.2018.06.023 30392627 PMC6220711

[aur2498-bib-0015] Frazier, T. W. , Strauss, M. , Klingemier, E. W. , Zetzer, E. E. , Hardan, A. Y. , Eng, C. , & Youngstrom, E. A. (2017). A meta‐analysis of gaze differences to social and nonsocial information between individuals with and without autism. Journal of the American Academy of Child & Adolescent Psychiatry, 56(7), 546–555. 28647006 10.1016/j.jaac.2017.05.005 PMC5578719

[aur2498-bib-0016] Frazier, T. W. , & Youngstrom, E. A. (2006). Evidence‐based assessment of attention‐deficit/hyperactivity disorder: Using multiple sources of information. Journal of the American Academy of Child & Adolescent Psychiatry, 45(5), 614–620. 16670656 10.1097/01.chi.0000196597.09103.25

[aur2498-bib-0017] Frazier, T. W. , Youngstrom, E. A. , Naugle, R. I. , Haggerty, K. A. , & Busch, R. M. (2007). The latent structure of cognitive symptom exaggeration on the Victoria Symptom Validity Test. Archives of Clinical Neuropsychology, 22(2), 197–211. 17276652 10.1016/j.acn.2006.12.007

[aur2498-bib-0018] Ganz, M. L. (2007). The lifetime distribution of the incremental societal costs of autism. Archives of Pediatrics & Adolescent Medicine, 161(4), 343–349. 17404130 10.1001/archpedi.161.4.343

[aur2498-bib-0019] Geddes, D. (2020). Upstate research leads to quadrant biosciences' release of first epigenetic test for autism Retrieved from https://www.upstate.edu/news/articles/2020/2020-01-02-epigenetic.php

[aur2498-bib-0020] Granpeesheh, D. , Tarbox, J. , & Dixon, D. R. (2009). Applied behavior analytic interventions for children with autism: A description and review of treatment research. Annals of Clinical Psychiatry, 21(3), 162–173. 19758537

[aur2498-bib-0021] Green, J. , Pickles, A. , Pasco, G. , Bedford, R. , Wan, M. W. , Elsabbagh, M. , Slonims, V. , Gliga, T. , Jones, E. , Cheung, C. , Charman, T. , Johnson, M. , & Cheung, C. (2017). Randomised trial of a parent‐mediated intervention for infants at high risk for autism: Longitudinal outcomes to age 3years. Journal of Child Psychology and Psychiatry, 58(12), 1330–1340. 10.1111/jcpp.12728 28393350 PMC5724485

[aur2498-bib-0022] Guyatt, G. , Rennie, D. , Meade, M. , & Cook, D. (2002). Users' guides to the medical literature: A manual for evidence‐based clinical practice (Vol. 706). AMA Press.

[aur2498-bib-0023] Hardan, A. Y. , Gengoux, G. W. , Berquist, K. L. , Libove, R. A. , Ardel, C. M. , Phillips, J. , Frazier, T. W. , & Minjarez, M. B. (2015). A randomized controlled trial of pivotal response treatment group for parents of children with autism. Journal of Child Psychology and Psychiatry, 56(8), 884–892. 10.1111/jcpp.12354 25346345

[aur2498-bib-0024] Hicks, S. D. , Ignacio, C. , Gentile, K. , & Middleton, F. A. (2016). Salivary miRNA profiles identify children with autism spectrum disorder, correlate with adaptive behavior, and implicate ASD candidate genes involved in neurodevelopment. BMC Pediatrics, 16, 52. 10.1186/s12887-016-0586-x 27105825 PMC4841962

[aur2498-bib-0025] Hicks, S. D. , Rajan, A. T. , Wagner, K. E. , Barns, S. , Carpenter, R. L. , & Middleton, F. A. (2018). Validation of a salivary RNA test for childhood autism spectrum disorder. Front Genetics, 9, 534. 10.3389/fgene.2018.00534 PMC6237842 30473705

[aur2498-bib-0026] Hicks, S. D. , Uhlig, R. , Afshari, P. , Williams, J. , Chroneos, M. , Tierney‐Aves, C. , Wagner, K. , & Middleton, F. A. (2018). Oral microbiome activity in children with autism spectrum disorder. Autism Research, 11(9), 1286–1299. 30107083 10.1002/aur.1972 PMC7775619

[aur2498-bib-0027] Hirvikoski, T. , Mittendorfer‐Rutz, E. , Boman, M. , Larsson, H. , Lichtenstein, P. , & Bölte, S. (2016). Premature mortality in autism spectrum disorder. The British Journal of Psychiatry, 208(3), 232–238. 26541693 10.1192/bjp.bp.114.160192

[aur2498-bib-0028] Howard, J. S. , Sparkman, C. R. , Cohen, H. G. , Green, G. , & Stanislaw, H. (2005). A comparison of intensive behavior analytic and eclectic treatments for young children with autism. Research in Developmental Disabilities, 26(4), 359–383. 15766629 10.1016/j.ridd.2004.09.005

[aur2498-bib-0029] Howlin, P. , Magiati, I. , & Charman, T. (2009). Systematic review of early intensive behavioral interventions for children with autism. American Journal on Intellectual and Developmental Disabilities, 114(1), 23–41. 19143460 10.1352/2009.114:23;nd41

[aur2498-bib-0030] Hyman, S. L. , Levy, S. E. , & Myers, S. M. (2020). Identification, evaluation, and management of children with autism spectrum disorder. Pediatrics, 145(1), e20193447. 10.1542/peds.2019-3447. 31843864

[aur2498-bib-0031] Jacobson, J. W. , Mulick, J. A. , & Green, G. (1998). Cost–benefit estimates for early intensive behavioral intervention for young children with autism—General model and single state case. Behavioral Interventions: Theory & Practice in Residential & Community‐Based Clinical Programs, 13(4), 201–226.

[aur2498-bib-0032] Jenkins, M. M. , Youngstrom, E. A. , Youngstrom, J. K. , Feeny, N. C. , & Findling, R. L. (2012). Generalizability of evidence‐based assessment recommendations for pediatric bipolar disorder. Psychological Assessment, 24(2), 269–281. 22004538 10.1037/a0025775 PMC3752420

[aur2498-bib-0033] Johnson, C. P. , & Myers, S. M. (2007). Identification and evaluation of children with autism spectrum disorders. Pediatrics, 120(5), 1183–1215. 17967920 10.1542/peds.2007-2361

[aur2498-bib-0034] Kasari, C. , Paparella, T. , Freeman, S. , & Jahromi, L. B. (2008). Language outcome in autism: Randomized comparison of joint attention and play interventions. Journal of Consulting and Clinical Psychology, 76(1), 125–137. 18229990 10.1037/0022-006X.76.1.125

[aur2498-bib-0035] KFF . (2020). Enhanced federal medical assistance percentage (FMAP) for CHIP. Retrieved from https://www.kff.org/other/state‐indicator/enhanced‐federal‐matching‐rate‐chip/?currentTimeframe=0&sortModel=%7B%22colId%22:%22Location%22,%22sort%22:%22asc%22%7D

[aur2498-bib-0036] Landa, R. J. (2018). Efficacy of early interventions for infants and young children with, and at risk for, autism spectrum disorders. International Review of Psychiatry, 30(1), 25–39. 29537331 10.1080/09540261.2018.1432574 PMC6034700

[aur2498-bib-0037] Leigh, J. P. , & Du, J. (2015). Brief report: Forecasting the economic burden of autism in 2015 and 2025 in the United States. Journal of Autism and Developmental Disorders, 45(12), 4135–4139. 26183723 10.1007/s10803-015-2521-7

[aur2498-bib-0038] Lovaas, O. I. (1987). Behavioral treatment and normal educational and intellectual functioning in young autistic children. Journal of Consulting and Clinical Psychology, 55(1), 3–9. 3571656 10.1037//0022-006x.55.1.3

[aur2498-bib-0039] Makino, A. , Wong, P. Y. , King, G. , Hartman, L. , & Penner, M. (2017). Parent perspectives and perceptions of autism spectrum disorder diagnosis: a scoping review. Paediatrics & Child Health, 22(Suppl 1), e9–e9. 10.1093/pch/pxx086.020

[aur2498-bib-0040] Medicaid.gov . (2019). Early and periodic screening diagnostic and treatment. Retrieved from https://www.medicaid.gov/medicaid/benefits/epsdt/index.html

[aur2498-bib-0041] Mohammadzaheri, F. , Koegel, L. K. , Rezaee, M. , & Rafiee, S. M. (2014). A randomized clinical trial comparison between pivotal response treatment (PRT) and structured applied behavior analysis (ABA) intervention for children with autism. Journal of Autism and Developmental Disorders, 44(11), 2769–2777. 24840596 10.1007/s10803-014-2137-3 PMC4194254

[aur2498-bib-0042] Musumeci, M. , & Chidambaram, P. (2019). Medicaid's role for children with special health care needs: A look at eligibility, services, and spending. *Kaiser Family Foundation*.

[aur2498-bib-0043] National Research Council Division of Behavioral and Social Sciences Education . (2001). Educating children with autism.

[aur2498-bib-0044] Oswald, D. P. , Haworth, S. M. , Mackenzie, B. K. , & Willis, J. H. (2017). Parental report of the diagnostic process and outcome: ASD compared with other developmental disabilities. Focus on Autism and Other Developmental Disabilities, 32(2), 152–160.

[aur2498-bib-0045] Parrish, T. , Harr, J. , Anthony, J. , Merickel, A. , & Esra, P. (2003). State special education finance systems, 1999–2000. Part I. American Institutes for Research.

[aur2498-bib-0046] Peacock, G. , Amendah, D. , Ouyang, L. , & Grosse, S. D. (2012). Autism spectrum disorders and health care expenditures: The effects of co‐occurring conditions. Journal of Developmental & Behavioral Pediatrics, 33(1), 2–8. 22157409 10.1097/DBP.0b013e31823969de

[aur2498-bib-0047] Peters‐Scheffer, N. , Didden, R. , Korzilius, H. , & Matson, J. (2012). Cost comparison of early intensive behavioral intervention and treatment as usual for children with autism spectrum disorder in The Netherlands. Research in Developmental Disabilities, 33(6), 1763–1772. 22705454 10.1016/j.ridd.2012.04.006

[aur2498-bib-0048] Peters‐Scheffer, N. , Didden, R. , Korzilius, H. , & Sturmey, P. (2011). A meta‐analytic study on the effectiveness of comprehensive ABA‐based early intervention programs for children with autism spectrum disorders. Research in Autism Spectrum Disorders, 5(1), 60–69. 10.1016/j.rasd.2010.03.011

[aur2498-bib-0049] Pierce, K. , Conant, D. , Hazin, R. , Stoner, R. , & Desmond, J. (2011). Preference for geometric patterns early in life as a risk factor for autism. Archives of General Psychiatry, 68(1), 101–109. 20819977 10.1001/archgenpsychiatry.2010.113 PMC4894313

[aur2498-bib-0050] Reichow, B. , Hume, K. , Barton, E. E. , & Boyd, B. A. (2018). Early intensive behavioral intervention (EIBI) for young children with autism spectrum disorders (ASD). The Cochrane Database of Systematic Reviews, 5(5), CD009260. 10.1002/14651858.CD009260.pub3 29742275 PMC6494600

[aur2498-bib-0051] Sackett, D. , Richardson, W. , Rosenberg, W. , & Haynes, R. (2000). Evidence‐based medicine: How to practice and teach EBM. Churchill Livingstone Inc.

[aur2498-bib-0052] Shimabukuro, T. T. , Grosse, S. D. , & Rice, C. (2008). Medical expenditures for children with an autism spectrum disorder in a privately insured population. Journal of Autism and Developmental Disorders, 38(3), 546–552. 17690969 10.1007/s10803-007-0424-y

[aur2498-bib-0053] Siu, A. L. , Bibbins‐Domingo, K. , Grossman, D. C. , Baumann, L. C. , Davidson, K. W. , Ebell, M. , García, F. A. , Gillman, M. , Herzstein, J. , Kemper, A. R. , Krist, A. H. , Kurth, A. E. , Owens, D. K. , Phillips, W. R. , W. R., M. G. , & Kemper, A. R. (2016). Screening for autism spectrum disorder in young children: US preventive services task force recommendation statement. JAMA, 315(7), 691–696. 10.1001/jama.2016.0018 26881372

[aur2498-bib-0054] U.S. Department of the Treasury . (2020). Daily treasury yield curve rates. Retrieved from https://www.treasury.gov/resource-center/data-chart-center/interest-rates/pages/textview.aspx?data=yield

[aur2498-bib-0055] Wagner, K. E. , McCormick, J. B. , Barns, S. , Carney, M. , Middleton, F. A. , & Hicks, S. D. (2019). Parent perspectives towards genetic and epigenetic testing for autism spectrum disorder. Journal of Autism and Developmental Disorders, 50(9), 3114–3125. 10.1007/s10803-019-03990-6 PMC6755071 30903561

[aur2498-bib-0056] Wiggins, L. D. , Durkin, M. , Esler, A. , Lee, L. C. , Zahorodny, W. , Rice, C. , Yeargin‐Allsopp, M. , Dowling, N. F. , Hall‐Lande, J. , Morrier, M. J. , Christensen, D. , Shenouda, J. , & Morrier, M. J. (2020). Disparities in documented diagnoses of autism Spectrum disorder based on demographic, individual, and service factors. Autism Research, 13(3), 464–473. 10.1002/aur.2255 31868321 PMC7521364

[aur2498-bib-0057] Youngstrom, E. A. , Van Meter, A. , Frazier, T. W. , Hunsley, J. , Prinstein, M. J. , Ong, M. L. , & Youngstrom, J. K. (2017). Evidence‐based assessment as an integrative model for applying psychological science to guide the voyage of treatment. Clinical Psychology: Science and Practice, 24(4), 331–363.

