# Supplementary material for: Evidence‐based use of scalable biomarkers to increase diagnostic efficiency and decrease the lifetime costs of autism
Source: Autism Res. 2021 Mar 8;14(6):1271–83. doi: 10.1002/aur.2498 (PMC8251791; doi:10.1002/aur.2498)
Supplement: Supplementary file 1 — Appendix S1: Avalere Health Validation [file AUR-14-1271-s003.pdf]

To: Quadrant Biosciences

From: Chris Sloan, Tim Epple

Date: March 10, 2020

Re: Validation of Studies Presented and Cost Savings Estimates Related to Autism Spectrum Disorder (ASD) Diagnostics

---

Overview

Avalere Health was commissioned by Quadrant Biosciences to perform a validation exercise of their manuscript entitled *Evidence-based use of scalable biomarkers to increase diagnostic efficiency and decrease the lifetime costs of autism*. The paper and underlying analysis attempt to estimate the potential cost savings stemming from the introduction of an early-age, objective diagnostic test to the Autism Spectrum Disorder (ASD) clinical assessment process.

Avalere conducted this validation exercise in two parts. First, a review of the paper provided by Quadrant Biosciences focused on validating the citations presented in the manuscript bibliography, as well as their overall import to the findings of the study. Second, Avalere evaluated the appropriateness of the quantitative methodology employed by Quadrant to estimate the cost savings, including a detailed examination of Quadrant's model and its underlying formulae and inputs. As part of the validation process, Avalere provided Quadrant Biosciences with a series of key questions and methodological notes , which were incorporated into Quadrant's analysis and underlying model. A description of Avalere's validation process and conclusions are provided in subsequent sections.

Validation of Studies Presented in the Analysis

Avalere performed a literature review and assessment of the clinical evidence cited by Quadrant Biosciences as part of their ASD market analysis and manuscript. To validate the studies presented in the paper provided by Quadrant Biosciences, Avalere segmented the citations into three (3) key focus areas, including studies related to 1) the accuracy of diagnostic tools for ASD; 2) identifying the diagnostic gap that currently exists in ASD treatment; and 3) studies related to the cost savings that could be derived from earlier identification and treatment of ASD in children through early intensive behavior intervention (EIBI). Across all segments, Avalere identified the journal, recency of publication, type of study, role of the study within the paper, sample size, age of the sample, and the country where the study was conducted. Avalere then identified specific focus areas within each segment.

Diagnostic Accuracy

In this segment, Avalere identified eight (8) studies that address the diagnostic accuracy of saliva and gaze-based ASD diagnostics. When assessing diagnostic accuracy, Avalere focused on studies presenting diagnostic effectiveness measured using Area Under the Curve (AUC) as a measure of model performance. Avalere confirmed the validity of the AUC across Frazier et al (2018), Hicks et al (2018), Frazier et al (2016), and Hicks et al (2016). Avalere also assessed whether the studies focused on the addressable population for early diagnosis of ASD. All identified studies included children, but they were not inherently focused on children aged 18-24 months, which Quadrant's paper states is the recommended screening age currently for children with ASD. Importantly, these studies were assessed against clinical and societal guidelines. Avalere was able to positively confirm the validity, clinical relevance, and cited extrapolations of the studies utilized by Quadrant with respect to diagnostic accuracy.

Diagnostic Gap

In this segment, Avalere identified nine (9) studies that address the diagnostic gap that currently exists in ASD treatment. These studies also focused on the importance and validity of EIBI which contributes to potential societal cost-savings. Avalere focused on the population size and the age of the population in the individual studies, noting that 5 of the studies included children aged 0-24 months, a key population for early EIBI. These studies validated the focus on benefits associated with early intensive behavioral interventions among 0-24 months old individuals with ASD.

Cost Savings

In this segment, Avalere identified ten (10) studies focused on the cost of ASD treatment, as well as the role earlier treatment and intervention contribute to potential societal cost savings. Again, Avalere focused on the age of individuals assessed and noted that seven of the 10 studies included children aged 18-24 months. Avalere also noted that the most impactful studies Peacock et al (2012) and Buescher et al (2014) assessed both outpatient and inpatient costs. Avalere further identified that across all 10 studies, the clinic, school, and home settings were assessed, ensuring all common settings of care were identified. Avalere was able to confirm the validity and relevance of citation of the studies utilized by Quadrant regarding cost-savings from EIBI.

Societal Cost Savings Estimates Validation

To validate the underlying calculations and analyses that inform the results of the paper, Avalere conducted a comprehensive model validation of the formulas and inputs to ensure that the results were methodologically appropriate. This process involved testing each of the documented formulae for accuracy, ensuring that appropriate discounting and inflation metrics were used for sources providing data prior to 2020, pressure testing assumptions, and varying key inputs to determine those that are most crucial to the underlying findings.

Copyright ©2020. Avalere Health LLC. All Rights Reserved.

The analysis is based on two separate estimates, one for educational cost savings and one for medical cost savings, that estimate the total costs for each grouping, by age, for a baseline population compared to a population with significant utilization of EIBI. EIBI is assumed to be administered for 3 years and between the ages 2 to 4, with associated benefits beginning at age 5. Importantly, the model does not attempt to detail any EIBI utilization in the baseline, but rather assumes that the baseline population is representative of individuals with ASD who do not receive EIBI, as described in the literature. Moreover, the model does not account for potential barriers to EIBI access, including distribution across all geographic areas and potential financial barriers for families.

To further ensure validation of the findings, Avalere performed a sensitivity analysis on the key inputs driving the estimates of societal cost savings due to EIBI. Avalere conducted a deterministic sensitivity analysis, explicitly varying four (4) key inputs that were identified in the review of the literature and regarded, by Avalere, as foundational to the cost savings estimation process. These key inputs included:

1. Epigenetic diagnostic ROC AUC
2. The distribution of individuals with ASD after EIBI across regular development, special education, and intensive special education
3. The distribution of individuals with ASD after EIBI by associated other medical comorbidities and/or intellectual disabilities
4. EIBI costs per year

The results are presented below in Table 1, detailing the findings of the sensitivity analysis. Of the inputs tested, progress made by individuals with ASD (e.g. regular development, special education, and intensive education) was the most sensitive input, swinging the relative total cost savings from -32% to 46% across the low and high sensitivity parameters, demonstrating that these inputs assumption are substantially driving the results. In like manner, the associated medical costs after EIBI and patient distribution sensitivity analyses leads to changes the relative results by approximately -33% to 33%.

Importantly, across all of the key inputs tested in the sensitivity analysis, the results consistently generated medical and educational cost savings ranging from $16B to $36B for all individuals with ASD over their lifetimes. Subsequently, Avalere concludes that the model is directionally correct within acceptable variation in its conclusion that, under the assumptions detailed in the paper and cited literature, increasing the use of EIBI will lead to societal cost savings.

Conclusion

Based on the results of the analysis conducted to validate the findings from the paper entitled *Evidence-based use of scalable biomarkers to increase diagnostic efficiency and decrease the lifetime costs of autism*, Avalere can confirm that the underlying citations and methodology are analytically sound and directionally correct within the framework of the assumptions stated in the paper.

Copyright ©2020. Avalere Health LLC. All Rights Reserved.

Avalere's analysis and methodological conclusions are based on the version of the underlying paper analyzed as of March 10, 2020. Avalere's copy of the paper from this date is available upon request.

Copyright ©2020. Avalere Health LLC. All Rights Reserved.

Table 1: Sensitivity Analysis of Key Inputs

	Sensitivity Parameters			Education Cost Savings			Medical Cost Savings			Total Cost Savings		
**Variable Inputs**	**Low**	**Model**	**High**	**Low**	**Model**	**High**	**Low**	**Model**	**High**	**Low**	**Model**	**High**
Epigenetic Diagnostic ROC AUC	60%	86%	97%	$9,326,557,899	$13,368,066,321	$15,077,935,269	$7,807,591,861	$11,190,881,667	$12,622,273,508	$17,134,149,759	$24,558,947,988	$27,700,208,777
Distribution of ASD Patients After EIBI												
Regular Development	17%	29%	49%									
Special Education	22%	34%	46%	$5,605,987,176	$13,368,066,321	$24,579,917,295	$11,190,881,667	$11,190,881,667	$11,190,881,667	$16,796,868,843	$24,558,947,988	$35,770,798,962
Intensive Special Education	61%	37%	5%									
Associated Medical Costs After EIBI												
ASD without Other Medical Conditions	19%	29%	39%									
ASD with Co-morbid Conditions	50%	40%	30%	$13,368,066,321	$13,368,066,321	$13,368,066,321	$2,999,096,724	$11,190,881,667	$19,382,666,610	$16,367,163,046	$24,558,947,988	$32,750,732,931
ASD with Intellectual Disability	31%	31%	31%									
EIBI Cost Per Year	$ 90,000	$ 45,000	$ 22,500	$13,368,066,321	$13,368,066,321	$13,368,066,321	$7,636,550,437	$11,190,881,667	$12,968,047,282	$21,004,616,758	$24,558,947,988	$26,336,113,603
