# Supplementary material for: Evidence‐based use of scalable biomarkers to increase diagnostic efficiency and decrease the lifetime costs of autism
Source: Autism Res. 2021 Mar 8;14(6):1271–83. doi: 10.1002/aur.2498 (PMC8251791; doi:10.1002/aur.2498)
Supplement: Supplementary file 2 — Appendix S2: Cost Savings Analysis Methodology [file AUR-14-1271-s002.docx]

Specific Calculations. Using the assumptions provided, cost savings estimates were calculated as follows:

Annual Cost Savings = ASDN (pvECS + pvMRCS), where:

ASDN = number of children in the US who will be diagnosed with ASD each year based on current prevalence statistics (estimated to be 64,209);

pvECS = the present value of marginal educational cost savings per ASD child; and

pvMRCS = the present value of marginal medical and residential cost savings per ASD child.

To begin, marginal educational and medical cost estimates for 2020 are calculated from 2012 costs using the published annual rates of inflation (for example, $100 of educational expenses in 2012 is estimated to be $103.57 in 2013, $107.27 in 2014… and $129.33 in 2020; similarly, $100 of medical and residential care expenses in 2012 is estimated to be $122.18 in 2020).

The present value of marginal educational cost savings is calculated as follows:

pvECS =$\sum_{n=4}^{20} [(EIn(\mathrm{SE}*8\%+ISE*81\%)-(SE*34\%+ISE*37\%))/(1+YLD)]^n$ where:

EI = annual inflation adjustment multiplier for education costs (for analysis year 1, this is 1.0374, for analysis year 2, this is 1.0762, etc.);

SE = marginal special education cost estimates for 2020;

ISE = marginal intensive special education cost estimates for 2020;

YLD = annual discount rate (30-year UST); and

*n* = analysis year.

The present value of marginal medical and residential cost savings is calculated as follows:

pvMRCS =$\sum_{n=4}^{53} [(MIn(ASDN-ASDE))/(1+YLD)]^n-\sum_{n=1}^{3} [(MIn(EIBI)/(1+YLD)]^n$ where:

MI = annual inflation adjustment multiplier for medical and residential care costs (for analysis year 1, this is 1.0302, for analysis year 2, this is 1.0613, etc.);

ASDN = estimated 2020 marginal medical and residential cost estimates for individuals without access to early intensive behavioral interventions (see below);

ASDE = estimated 2020 marginal medical and residential cost estimates for individuals with access to early intensive behavioral interventions (see below);

EIBI = cost of early intensive behavioral interventions (estimated $45,000);

YLD = annual discount rate (30-year UST); and

*n* = analysis year.

For any analysis year, ASDN and ASDE are calculated as follows:

ASDN = MMRC*a**58%*CMCM, and

ASDE = MMRC*a**40%*CMCM, where:

MMRC = estimated 2020 marginal medical and residential care cost estimates for ASD individuals without intellectual disabilities based on age (see Table 4 for 2012 marginal costs by age);

CMCM = average cost multiplier for ASD children with co-morbid conditions such as ADHD and Epilepsy, estimated to be 1.48(Peacock et al., 2012) and

*a* = age of the patient in the analysis year, or *n*+1.

*Note: The medical and residential costs associated with the estimated 31% of ASD children with intellectual disabilities are conservatively assumed to be the same regardless of access to early intensive behavioral interventions; further, those children with ASD who ultimately become functionally indistinguishable from their peers are assumed to have normal (non-ASD) medical expenses thereafter. Accordingly, these costs are ignored in this marginal cost/benefit analysis. For those children with ASD who experience meaningful but more moderate gains and those children who experience minimal gains but do not have an intellectual disability, we assume the existence of other co-morbid medical conditions.*
