# Supplementary material for: Evidence‐based use of scalable biomarkers to increase diagnostic efficiency and decrease the lifetime costs of autism
Source: Autism Res. 2021 Mar 8;14(6):1271–83. doi: 10.1002/aur.2498 (PMC8251791; doi:10.1002/aur.2498)
Supplement: Supplementary file 3 [file AUR-14-1271-s004.docx]

	Sensitivity Parameters			Education Savings ($ billions)			Medicaid Savings ($ billions)			Total Public Savings ($ billions)		
**Variable Inputs**	**Low**	**Model**	**High**	**Low**	**Model**	**High**	**Low**	**Model**	**High**	**Low**	**Model**	**High**
Epigenetic Diagnostic ROC AUC	60%	86%	97%	$ 9.33	$ 13.37	$ 15.08	$ 7.81	$ 11.19	$ 12.62	$ 17.13	$ 24.56	$ 27.70
Distribution of ASD Patients After EIBI												
Regular Development	17%	29%	49%									
Special Education	22%	34%	46%	$ 5.61	$ 13.37	$ 24.58	$ 11.19	$ 11.19	$ 11.19	$ 16.80	$ 24.56	$ 35.77
Intensive Special Education	61%	37%	5%									
Associated Medical Costs After EIBI												
ASD without Other Medical Conditions	19%	29%	39%									
ASD with Co-morbid Conditions	50%	40%	30%	$ 13.37	$ 13.37	$ 13.37	$ 3.00	$ 11.19	$ 19.38	$ 16.37	$ 24.56	$ 32.75
ASD with Intellectual Disability	31%	31%	31%									
EIBI Cost Per Year	$ 90,000	$ 45,000	$ 22,500	$ 13.37	$ 13.37	$ 13.37	$ 7.64	$ 11.19	$ 12.97	$ 21.00	$ 24.56	$ 26.34
